# Use of Self-Selected Postures to Regulate Multi-Joint Stiffness During Unconstrained Tasks

**DOI:** 10.1371/journal.pone.0005411

**Published:** 2009-05-01

**Authors:** Randy D. Trumbower, Matthew A. Krutky, Bing-Shiang Yang, Eric J. Perreault

**Affiliations:** 1 Sensory Motor Performance Program, Rehabilitation Institute of Chicago, Chicago, Illinois, United States of America; 2 Department of Biomedical Engineering, Northwestern University, Evanston, Illinois, United States of America; 3 Department of Mechanical Engineering, National Chiao Tung University, Hsinchu, Taiwan; 4 Brain Research Center, National Chiao Tung University, Hsinchu, Taiwan; 5 Department of Physical Medicine and Rehabilitation, Northwestern University, Chicago, Illinois, United States of America; The University of Western Ontario, Canada

## Abstract

**Background:**

The human motor system is highly redundant, having more kinematic degrees of freedom than necessary to complete a given task. Understanding how kinematic redundancies are utilized in different tasks remains a fundamental question in motor control. One possibility is that they can be used to tune the mechanical properties of a limb to the specific requirements of a task. For example, many tasks such as tool usage compromise arm stability along specific directions. These tasks only can be completed if the nervous system adapts the mechanical properties of the arm such that the arm, coupled to the tool, remains stable. The purpose of this study was to determine if posture selection is a critical component of endpoint stiffness regulation during unconstrained tasks.

**Methodology/Principal Findings:**

Three-dimensional (3D) estimates of endpoint stiffness were used to quantify limb mechanics. Most previous studies examining endpoint stiffness adaptation were completed in 2D using constrained postures to maintain a non-redundant mapping between joint angles and hand location. Our hypothesis was that during unconstrained conditions, subjects would select arm postures that matched endpoint stiffness to the functional requirements of the task. The hypothesis was tested during endpoint tracking tasks in which subjects interacted with unstable haptic environments, simulated using a 3D robotic manipulator. We found that arm posture had a significant effect on endpoint tracking accuracy and that subjects selected postures that improved tracking performance. For environments in which arm posture had a large effect on tracking accuracy, the self-selected postures oriented the direction of maximal endpoint stiffness towards the direction of the unstable haptic environment.

**Conclusions/Significance:**

These results demonstrate how changes in arm posture can have a dramatic effect on task performance and suggest that postural selection is a fundamental mechanism by which kinematic redundancies can be exploited to regulate arm stiffness in unconstrained tasks.

## Introduction

Many functional tasks, such as the use of hand tools, compromise the stability of the arm in a specific direction [1]. For example, use of a screwdriver compromises limb stability in directions orthogonal to the long axis of the tool, toward which it tends to topple when exerting forces against the head of a screw. This task only can be performed if the nervous system adapts the mechanical properties of the arm such that the arm, coupled to the tool, remains stable. One way to quantify arm stability during such postural tasks is through estimates of endpoint stiffness, which characterizes the static mechanics of the limb as seen at the point of contact with the environment [2]. Hogan [3] first proposed that endpoint stiffness may be regulated specifically to compensate for such instabilities.

There are a number of ways by which endpoint stiffness can be regulated. Changes in limb posture have a profound effect on the orientation of maximal stiffness [2], [4]. At a fixed posture, stiffness can be regulated through changes in muscle activation. These changes in activation can occur via feedforward changes in co-contraction [5], [6] or through changes in the sensitivity of reflex feedback [7]–[10]. Numerous studies have focused on how changes in muscle activation can lead to task-appropriate changes in limb stiffness, increasing limb stability and endpoint accuracy during both reaching and postural tasks [9], [11]–[14]. However, Milner suggested that these changes and their corresponding functional consequences can be small relative to those associated with changes in limb posture [15]. Because most studies examining the control of limb stiffness have constrained limb posture, it is unclear if posture selection is a critical component of endpoint stiffness regulation during more natural tasks in which the kinematic redundancies of the arm can be exploited to change posture without altering endpoint location.

The purpose of this study was to determine if posture selection is a critical component of endpoint stiffness regulation during unconstrained tasks. Our hypothesis was that subjects would select arm postures that matched endpoint stiffness to the functional requirements of the task. The hypothesis was tested during endpoint tracking tasks in which subjects interacted with unstable haptic environments, simulated using a three degrees of freedom (3DOF) robotic manipulator. The study had three specific goals. The first was to quantify how arm posture influenced tracking performance during interactions with unstable environments. The second was to determine if subjects self-selected similar postures when interacting with the same haptic environment. The third goal was to determine if the self-selected postures oriented the direction of maximal endpoint stiffness in a manner that would best compensate for the unstable nature of the haptic environment. Our results demonstrate how changes in arm posture can have a dramatic effect on task performance and suggest that postural selection is a fundamental mechanism by which arm stiffness is controlled in unconstrained tasks.

## Materials and Methods

### Ethics Statement

The protocol was approved by the Institutional Review Board of Northwestern University's Office for the Protection of Research Subjects (IRB#1322-001). All subjects gave written, informed consent and were free to withdraw at any time.

### Subjects

Nine subjects, 24 to 40 years of age (7 males and 2 females), participated in this study. Subjects had no history of neurological or orthopedic impairments of the upper limbs. Data were collected in two separate experimental sessions. All subjects participated in the first experiment and five subjects returned to the laboratory for the second.

### Equipment

**Figure 1:**
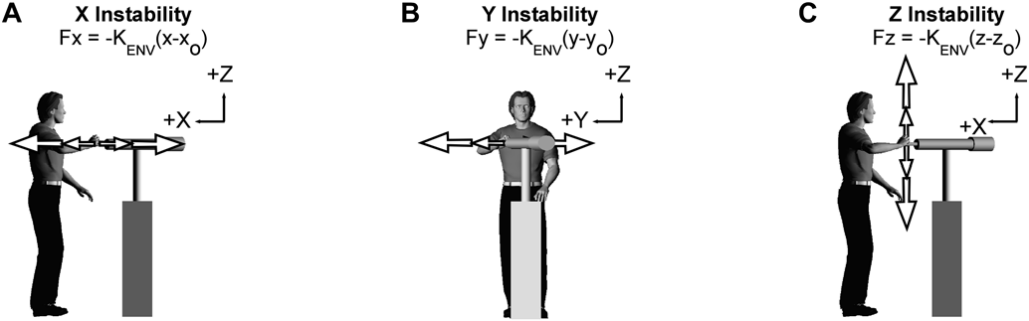
Experimental setup for tracking task. Subjects stood upright and used the arm to interact with unstable haptic environments oriented along the X (A), Y (B) or Z (C) measurement axes. During target tracking, movements were constrained to lie along these axes by 50 kN/m virtual walls.

Subjects interacted with a 3DOF robotic manipulator [HapticMaster; FCS Control Systems, The Netherlands; Figure 1A–1C] during both experiments. The robot uses an admittance control algorithm, allowing it to simulate a range of haptic environments [16]. It was used to simulate unstable haptic environments during the first set of experiments and as a position servo to perturb the limb for estimating endpoint stiffness in the second set of experiments. The robot was instrumented to measure endpoint displacements and forces, both which were recorded at 1.25 kHz. During the second set of experiments, endpoint displacement was redundantly measured using an optical motion analysis system [Optotrak 3020; Northern Digital, Waterloo, Ontario] with an accuracy of 0.1 mm. The optical tracking data were used to correct for small errors in the endpoint displacement measures obtained from the robot, due to compliance between the robot's end effector and its displacement sensors. The Optotrak tracks the motion of infrared LEDs, which were mounted on a rigid body attached to a wrist cast and used to monitor endpoint location. All optical data were collected at 250 Hz and later interpolated to 1.25 kHz to match the sampling rate of the robotic system. Data acquisition was synchronized between the two systems through the use of a common clock and trigger.

### Protocols

#### Endpoint tracking

The purpose of the first experiment was to quantify the influence of prescribed and self-selected arm postures on the ability to control hand position during interactions with unstable environments. We attempted to have subjects interact with the simulated environments under functionally relevant conditions by removing all physical restraints between the subject and the robot and by having subjects support the weight of their arm in all tasks. Subjects stood upright with the feet side-by-side during these experiments, with the shoulders parallel to the Y-axis (Figure 1). They interacted with the robot by grasping a plastic sphere attached to the endpoint. The center of the sphere was defined as the endpoint of the arm. The shoulder and elbow were unconstrained in these first experiments, requiring subjects to support the weight of the arm against gravity. The only postural constraint was that the hand always was positioned in the sagittal plane at the height of the sternum, so as to restrict the experimental degrees of freedom to a manageable number.

The haptic environment simulated in these experiments was primarily a “negative-stiffness” spring, acting along a line in 3D space. These environments were oriented into and away from the body in the sagittal plane (±X; Figure 1A), in the medial-lateral direction (±Y; Figure 1B), or in the vertical direction (±Z; Figure 1C). As subjects moved the position of their hand away (x, y, or z) from the neutral position (x_o_, y_o_, or z_o_), the robot pushed the hand further with a force (F) proportional to the distance between the hand and the neutral point, according to the equations displayed in Figure 1. The strength of the unstable haptic environments was −500 N/m for all subjects except two who could not maintain endpoint stability when interacting with an environment of this strength (Table 1). For directions orthogonal to the line of instability, the haptic environment was programmed to be rigid, having a stiffness of 50 kN/m. The haptic environment had a simulated mass of 5.0 kg and was critically damped in all directions; virtual stops were located at a distance of ±100 mm from the neutral position to ensure the safety of subjects. The negative stiffness values used in these experiments were greater than those reported in previous similar experiments [15], as was needed to challenge the subjects' ability to maintain stable endpoint positions. Two factors likely contributed to this need. The first is that subjects were required to support the weight of their limb during our experiments. The increased muscle activation associated with this task would increase endpoint stiffness beyond that in the supported conditions reported previously. The second factor is our use of an admittance controller. This controller required the simulated haptic environment to have mass as well as stiffness. These simulated inertial properties provided some resistance transient perturbations of posture, and also may have contributed to the increased negative stiffness required in these experiments.

**Table 1:** Haptic environments used during endpoint tracking.

Subject	Strength of Unstable Haptic Environment (N/m)		
	X	Y	Z
1*	−500	−500	−500
2*	−500	−500	−500
3*	−500	−500	−500
4	−500	−500	−500
5	−500	−500	−500
6*	−300	−300	−300
7*	−500	−400	−300
8	−500	−500	−500

* denotes that subject participated in endpoint stiffness experiment.

The subjects' task was to track specified endpoint target locations while interacting with the unstable haptic environments. The target was randomly positioned in one of three locations (−10 mm, 0 mm, and +10 mm) relative to the neutral point of the haptic environment. The target was held at each location for four seconds before it appeared at the next location. Each trial contained 19 target jumps, lasting for a total of 76 s. Subjects received visual feedback of endpoint and target location and were instructed to move to each target as rapidly and accurately as possible. Subjects were instructed to support the weight of their limb during these experiments and not to rely on the rigid walls orthogonal to the haptic instabilities. Visual feedback of endpoint forces was not provided during the tracking experiments, but a subsequent analysis indicated that subjects supported approximately 85% or their arm mass during tracking. The average forces exerted against the X, Y and Z constraints were 1.3±0.2 N, 0.0±0.0 N and −1.8±0.3 N, respectively (mean±SE).

To test the influence of arm posture on the ability to control the endpoint location, subjects performed the tracking task at four prescribed arm postures, then at one self-selected posture. The prescribed postures were chosen to examine the effect of hand position and shoulder abduction, the two unconstrained degrees of freedom, on tracking performance. For the prescribed postures the hand was located directly in front of the sternum at a distance of either 1/3 or 5/6 the length of the arm, and the shoulder was abducted to either ∼20° or ∼80°. The lesser angle was chosen to position the arm close to the trunk, while avoiding any contact; the greater angle was chosen as required to keep the forearm horizontal. In both cases, the available angles were limited by the constraint of keeping the hand in front of the sternum. Hand location was set with a resolution of 1 cm, as measured using a tape measure. Shoulder angles were set manually with a goniometer and then remeasured prior to the start of each experiment. The actual abduction angles were 21±2° and 71±3°. For the self-selected posture, subjects had their choice of hand location and shoulder abduction angle, with only two restrictions: (1) the hand was restricted to be in front of the sternum and (2) the arm was not allowed to touch the trunk or be lifted above the horizontal plane. The maintenance of consistent arm postures was monitored visually during the experiment by observing the height of the elbow relative to the starting position. Trials in which elbow height changed by more than ±2 cm were repeated.

Two consecutive endpoint tracking trials were repeated at each of the four prescribed postures, followed by two trials at the self-selected posture. Subjects were allowed as much time as needed to choose a self-selected posture, but once the posture was selected it was kept constant throughout the course of the data collection trials. Allowing subjects to stand during these tracking experiments made it easy for them to manipulate the available degrees of freedom and choose an appropriate self-selected posture. For each environment with which subjects interacted (X, Y, and Z), all five postures were tested consecutively before subjects interacted with a new environment. A total of 30 trials were performed (5 postures×2 trials×3 environments). The sequential orders of the prescribed postures and the haptic environments were randomized across subjects. A minimum of a two minute rest period was provided between successive trials to prevent fatigue.

#### Endpoint stiffness

The purpose of the second experiment was to quantify the orientation of maximal endpoint stiffness at the self-selected postures chosen during interactions with each of the three unstable environments (X, Y, and Z). For this purpose the robot was configured as a stiff position servo and used to apply 3D, stochastic perturbations to the endpoint of the arm. The perturbations were similar to those we have used previously [17], [18], having a standard deviation of 3.0 mm and frequency spectrum that was flat up to 5 Hz, beyond which it decayed at a rate of 40 dB/decade. Subjects were rigidly attached to the robot using a custom-fitted fiberglass cast. The cast extended ∼1/3 of the distance from the wrist to the elbow, fixing the wrist in the neutral position. The cast was mounted to a low mass, custom gimbal attached to the end of the manipulator, allowing the application of pure endpoint forces and no moments to the arm. The gimbal was instrumented with potentiometers that were used to provide subjects with visual feedback of arm posture so that a fixed posture could be maintained throughout each trial. The gimbal's center was positioned along the axis of the forearm, under the middle metacarpophalangeal joint, which we defined as the endpoint of the limb for these experiments. Subjects were seated during these experiments and harnessed at the shoulders and waist to an immobile chair. We chose to restrain subjects at the trunk and shoulders so that estimates of endpoint stiffness would characterize only the mechanical properties of the arm, not the net mechanical properties of the arm coupled to the unconstrained shoulder girdle and trunk [19], [20] since we only manipulated arm postures in these experiments.

**Figure 2:**
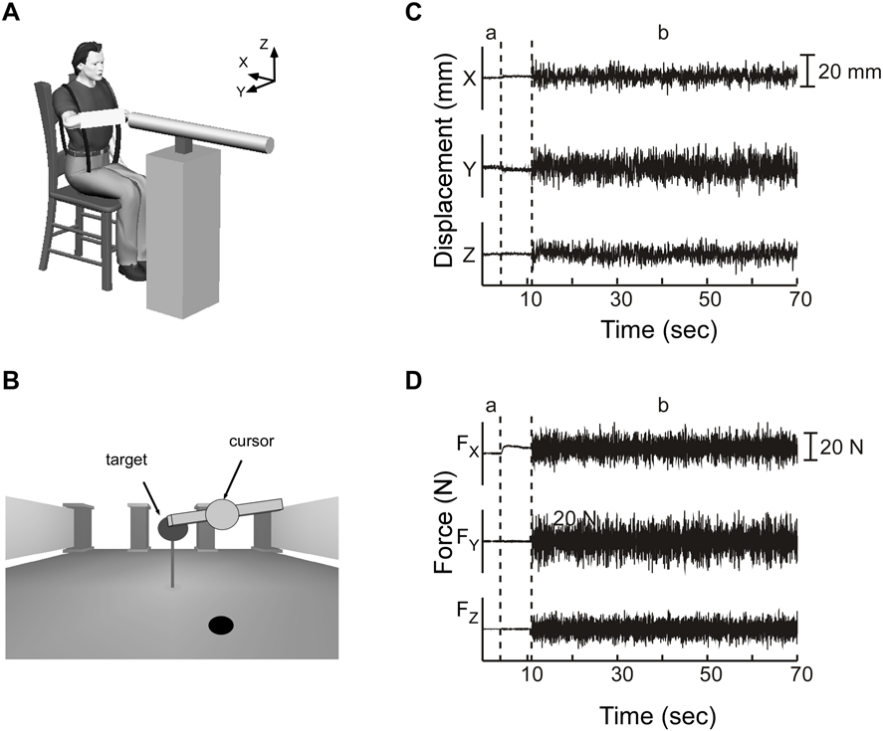
Stiffness estimation experiments. (A) Subjects were seated and strapped to a rigid chair in all stiffness estimation experiments. (B) Visual feedback of the three-dimensional target endpoint force and measured endpoint force was provided to the subject, as was the orientation of the arm. Arm orientation was displayed by the angle of a bar attached to the endpoint force cursor. (C) Endpoint displacements in the X, Y, and Z directions are shown for a typical stiffness estimation experiment. (D) The corresponding endpoint forces. In this trial, the subject was instructed to generate 10 N in the +X direction. Data from the first 2 seconds of each trial were used to record baseline values (‘a’). Afterwards, a visual cue instructed the subject to generate an endpoint force to match the target force. Once the target force was held steady, a stochastic perturbation was applied (‘b’), lasting for 60 s.

The subjects' task was to maintain a fixed arm posture while exerting a constant endpoint force, but not reacting to the perturbation. The endpoint force targets were ±5 N or ±10 N. These magnitudes were chosen to be similar to those encountered during the endpoint tracking tasks. The direction of the target forces was matched to the orientation of the haptic environment for which each posture was selected. For example, target forces were in the X direction when the subject was positioned in the posture they selected to interact with the environment unstable along the X direction. Real-time visual feedback of the 3D endpoint force was displayed to the subject (Figure 2B). The angle of shoulder elevation, which was the only unconstrained degree of freedom, also was displayed. During each trial (Figure 2C and 2D), subjects first supported the arm's weight against gravity for 2 s, after which a visual cue corresponding to the target force was presented. Once the target force was reached and held steady (within ±1 N) for 0.7 s, the robot applied a stochastic perturbation that lasted for 60 s. Only the final 55 s of each trial were analyzed, to avoid the small non-stationary corrective movements occasionally observed immediately following perturbation onset. Four trials were conducted for each combination of posture and endpoint force, resulting in a total of 48 trials for each subject (3 self-selected postures×4 forces×4 repetitions).

We were interested in determining how well the self-selected postures aligned the direction of maximal endpoint stiffness with the orientation of the unstable haptic environments. To interpret our results, we needed also to quantify the range of endpoint stiffness orientations that could be achieved by moving the arm throughout the range of postures allowed in these experiments. This was accomplished by estimating endpoint stiffness from two subjects at 9 prescribed postures spanning the allowable range. These corresponded to all combinations of three hand positions and three shoulder abduction angles: the hand positions were (1) as near and (2) as far as possible from the sternum, and (3) midway in between; the shoulder abduction angles were (1) full abduction (∼80°, keeping the forearm horizontal), (2) the arm nearly touching the trunk (∼20°), and (3) midway between (∼45°). Stiffness was estimated while subjects applied ±10 N in the X, Y, and Z directions at each posture. All other aspects of this experiment were identical to those described above.

We also ran a control experiment to determine the influence of pronation/supination on the orientation of maximal endpoint stiffness. It was difficult to accurately measure pronation/supination angles during the self-selected tracking experiments, and we were concerned that differences in pronation/supination between the tracking and stiffness experiments could have altered our results. Therefore, we quantified the influence of pronation/supination in two subjects by estimating endpoint stiffness with the forearm supinated to 45°, pronated to 45°, and in the neutral position for a single arm posture (elbow flexed to 90°; shoulder elevated to 70°). Target forces were 10 N along the X and Y axes. All other aspects of this control experiment were identical to those described above.

### Analysis

#### Endpoint tracking experiments

We hypothesized that arm posture would significantly affect each subject's ability to accurately control hand position during interactions with unstable environments. This was tested by quantifying the error with which subjects tracked the endpoint target, then comparing this error across the tested postures. Tracking error for each trial (T_ERR_) was quantified using the root mean square (RMS). Since our goal was to evaluate the ability to hold the hand at each of the stationary targets, not the ability to move between targets, only the final 3 s of the 4 s hold phases were used for the analysis. To facilitate comparing data across subjects and conditions, tracking errors at the 4 prescribed postures were normalized by the error at the self-selected posture for each environment. The influence of posture on tracking was assessed using linear mixed-effects model computed in R [21]. The independent factors in this analysis were hand position and shoulder abduction angle; subjects were treated as a random factor. All confidence intervals are reported as mean±standard error.

A secondary goal of the tracking experiment was to determine if subjects self-selected postures with similar hand positions and shoulder abduction angles when interacting with the same haptic environment. This was examined by plotting the relationship between the selected shoulder abduction angles and hand positions for each self-selected postures. Statistical comparisons of the postures selected for each haptic environment were obtained using a jackknife analysis [22] to account for potentially non-gaussian distributions. Significance levels of 0.05 were used for these numerically evaluated tests.

#### Endpoint stiffness experiments

**Figure 3:**
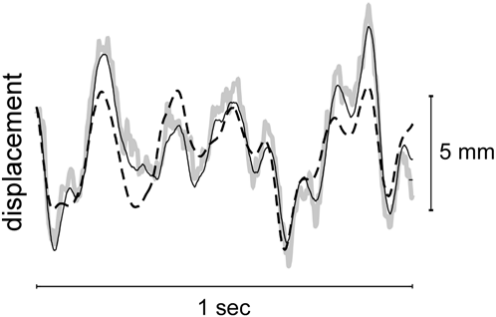
Refined estimate of endpoint displacement. An instrumental variable (IV) technique was used to increase the accuracy of the measured endpoint displacement. The figure shows typical data measured along the X axis. The displacement measured by the robot sensors is shown by the black dashed line; it differs from the true displacement due to compliance in the robot transmission. A noisy, but more accurate estimate was obtained using optical tracking (thick gray line). The combined estimate, obtained using instrumental variables, is shown by the solid black line.

Endpoint impedance completely describes the dynamic relationship between displacements applied to the hand and the forces generated in response. Endpoint stiffness is the static component of impedance and can be obtained from these more general estimates. Endpoint impedance was calculated from the endpoint position and force data collected during the stochastic perturbation experiments using nonparametric system identification techniques we have described previously [23]. Force data were utilized as recorded by the robot. The redundant displacements measured with the motion analysis system were used to account for compliance between the robot's displacement sensors and endpoint. To enhance the resolution of the optically recorded displacement data, we used an instrumental variables technique [24] to predict the optically measured endpoint displacements from the displacements estimated from the robot sensors. This technique could be used because the displacements estimated from the robot sensors were correlated with those measured by the motion analysis system, but not with the noise in the motion analysis data, which arose mainly from quantization errors. The resulting displacement data had reduced noise but were corrected to compensate for any transmission compliance (Figure 3). This led to a small (1.3±0.3%), but significant (paired t-test; t = 4.79; P<0.001), improvement in the ability to estimate the force response to the applied perturbations. The use of this technique also reduces the possibility for bias errors associated with noise on the input [25].

Because this study was concerned with postural control, we chose to focus on the static component of endpoint impedance, endpoint elasticity or stiffness. The dynamics equations describing impedance can be expressed in the frequency domain by Eq. 1, where *f* is frequency, *F_x_(f)*, *F_y_(f)*, *F_z_(f)* are the Fourier transforms of the endpoint forces along each measurement axis, *X(f)*, *Y(f)*, and *Z(f)* are the Fourier transforms of the endpoint displacements, and *H_ij_(f)* are the nine transfer functions relating displacements in the direction *j* to forces in the direction *i*. These transfer functions were estimated nonparametrically [23], and then parameterized by fitting a second order model with inertial, viscous, and elastic parameters. These fits were conducted over the frequency range of 0–10 Hz using least squares optimization, as we have described previously [18], [23]. This resulted in 3×3 matrices characterizing the endpoint inertia (*I*), viscosity (*B*) and stiffness (*K*).
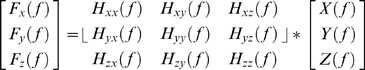
(1)


Three measurements were used to evaluate the quality of the estimated impedance models. First, we evaluated the nonparametric fit for each trial using the multiple correlation coefficient, R^2^, to characterize the relationship between the predicted and measured endpoint forces. Next, multiple coherence was used to determine the range of frequencies for which the linear transfer functions were appropriate [25]. Finally, we quantified how well the predicted endpoint force, obtained using the estimated *I*, *B*, and *K* parameters, approximated the actual force, again using R^2^.

Endpoint stiffness can be described graphically using an ellipsoid [2]. The long axis of the ellipsoid describes the direction in which the arm is most resistant to postural disturbances. The principal axes of the stiffness ellipsoid were calculated using singular value decomposition, as described by Gomi and Osu [26].

## Results

### Effect of Posture on Tracking Performance

**Figure 4:**
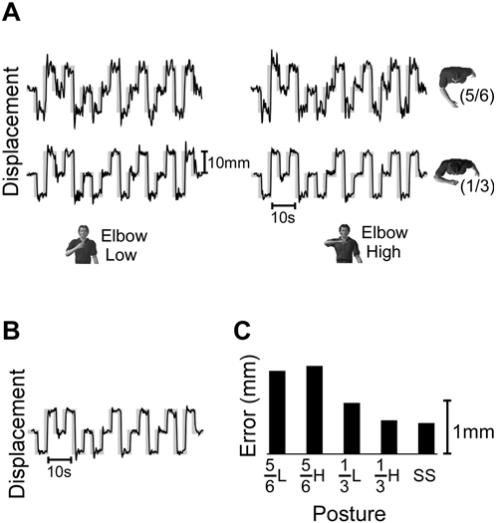
Endpoint tracking data collected during individual trials from a single subject. Data were collected during interactions with the Y instability. Black and gray lines correspond to the actual and target positions, respectively. (A) Data collected at the four prescribed postures, as indicated by the characters shown in each row and column. ‘Elbow low’ and ‘elbow high’ correspond to 25° and 90° of shoulder abduction, respectively. ‘1/3’ and ‘5/6’ correspond to hand position at 1/3 and 5/6 arm length from the sternum, respectively. (B) Endpoint tracking data from the same subject at the self-selected posture. (C) RMS tracking error for each of the trials presented in Figure 3A and 3B. Labels correspond to ‘hand position (1/3 or 5/6 arm length) +elbow height (Low or High)’ or the self-selected posture (‘SS’).

Arm posture had a strong influence on tracking error. This can be seen in Figure 4A, which displays typical endpoint position data for tracking performed during interactions with the Y instability. A single trial is shown for each prescribed posture. Trials in which endpoint position (thin black lines) closely follows the target (thick gray lines) correspond to trials with low error. For example, across the prescribed postures tracking error was lowest with the hand at 1/3 arm length from the sternum and the shoulder abducted to 90° (elbow high). Even lower tracking errors were recorded at the self-selected posture (Figure 4B), which for this subject and task corresponded to a normalized hand distance of ∼30% arm length from the sternum to the hand and a shoulder abduction angle of 71°. The tracking results for this subject are summarized in Figure 4C, which displays the average tracking error for each trial displayed in Figure 4A and 4B.

**Figure 5:**
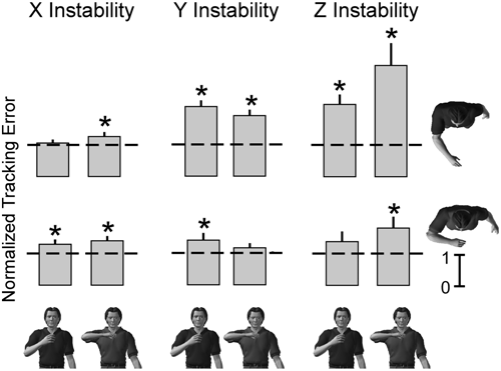
Group data for the endpoint tracking task. All data have been normalized to the tracking errors recorded at the self-selected posture; a normalized value of 1.0 is indicated by the dashed line. Characters depict the prescribed postures during interactions with each of the haptic environments. Tracking errors significantly greater than those measured at the self-selected posture are indicated by an * (p<0.05).

Across all subjects, tracking errors were lowest at the self-selected postures. This can be seen from the group data presented in Figure 5. In this figure, the tracking errors from each subject are normalized by those recorded at the self-selected posture for each environment. Across all conditions, the normalized tracking errors are never significantly less than 1, indicating that the greatest tracking accuracy was achieved at the self-selected postures.

The influence of arm posture on tracking accuracy varied with the directional characteristics of the environment. Posture had the greatest influence on tracking accuracy during interactions with the Z instability. The effects of both hand position and shoulder abduction were significant (t_position_ = 4.82, p_position_<0.001; t_shoulder_ = 3.73, p_shoulder_<0.001), but the interaction term was not (t_interaction_ = 1.47; p_interaction_ = 0.15). For the range of postures tested, changes in hand distance had a slightly larger effect (1.72±0.36) than changes in shoulder angle (1.24±0.33), although these differences did not reach statistical significance. Finally, all prescribed postures had a tracking error that was significantly greater than that at the self-selected posture (all t>3.44; all p<0.004), except for the posture corresponding to the hand held at 1/3 arm length from the body and the shoulder abducted to 25°.

A similar but less dramatic influence of arm posture was observed during interactions with the Y instability. Again, changes in both hand position and shoulder abduction had significant effects on tracking accuracy (t_position_ = 7.52, p_position_<0.001; t_shoulder_ = 2.67, p_shoulder_ = 0.01), while the interaction term was insignificant (t_interaction_ = 0.18; p_interaction_ = 0.86). Changes between the prescribed hand positions had a significantly larger effect (0.81±0.11) than that due to changes between the prescribed shoulder abduction angles (0.27±0.10) during interactions with the Y instability. The only prescribed posture that did not have significantly greater tracking errors than the self-selected postures was with the shoulder abducted to 90° and the hand at 1/3 arm length from the sternum; all others had larger tracking error (all t>3.04; all p<0.007) than the self-selected posture.

The least dramatic effect of posture on tracking accuracy occurred for interactions with the X instability. The influence of shoulder abduction on the observed tracking errors was small but significant (t_shoulder_ = 2.46, p_shoulder_ = 0.017), but the influence of hand position did not reach significance (t_position_ = 1.82, p_position_ = 0.074); the interaction term also did not reach significance (t_interaction_ = 0.64, p_interaction_ = 0.52). There was no significant difference between the influence of shoulder abduction (0.20±0.08) and hand position (0.17±0.09). Even with these small posture-dependent changes in tracking accuracy, tracking performance at most prescribed postures was significantly worse than that at the self-selected posture. The two were statistically indistinguishable only for the prescribed posture corresponding to the hand at 5/6 arm length from the sternum and the shoulder abducted to 25° (t = 0.86, p = 0.404). Tracking accuracy was worse at all other prescribed postures (all t>3.18; all p<0.006).

### Self-Selected Postures

**Figure 6:**
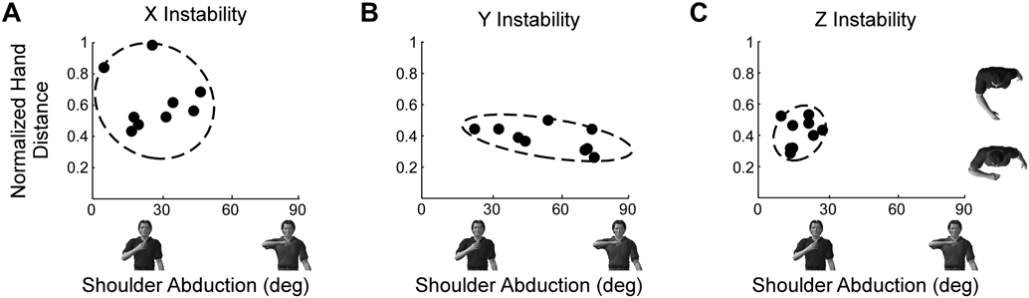
Self-selected postures during interactions with each of the haptic environments. Postures were chosen during interactions with unstable environments aligned to the X (A), Y (B) and Z (C) axes. Each filled circle corresponds to the posture chosen by a single subject. Dashed lines correspond to 90% confidence interval ellipses, computed from the covariance between shoulder angle and hand position [45]. The characters are placed at locations along horizontal and vertical axes corresponding to the posture that they depict.

The self-selected postures were different during interactions with each haptic environment. This can be seen in Figure 6, which summarizes the postures selected by all subjects. Shoulder abduction was larger during interactions with the Y-instability relative to that during interactions with the other two haptic environments. Hand distance from the sternum was greatest during interactions with the X-instability. No other comparisons reached statistical significance at the level of p<0.05.

Subjects tended to self-select similar postures when interacting with environments in which posture had a large effect on tracking accuracy. This can be seen by the variability of the self-selected postures for each of the haptic environments. Shoulder angle had the most dramatic effect on tracking accuracy during interactions with the Z instability and the variance of the self-selected postures was lowest during interactions with this haptic environment (p<0.05). There also was a tendency for reduced variability of the self-selected hand positions during interactions with the Z and Y instabilities, although this did not reach statistical significance when compared to the large variability observed during interactions with the X instability.

Although there was variability in the self-selected postures, the 90% confidence ellipsoids of the postures selected for each haptic environment each encompass one of the prescribed postures used during the tracking experiments. These are the same prescribed postures for which the tracking error was not significantly different than that at the self-selected posture (Figure 5).

### Estimates of Endpoint Stiffness

**Figure 7:**
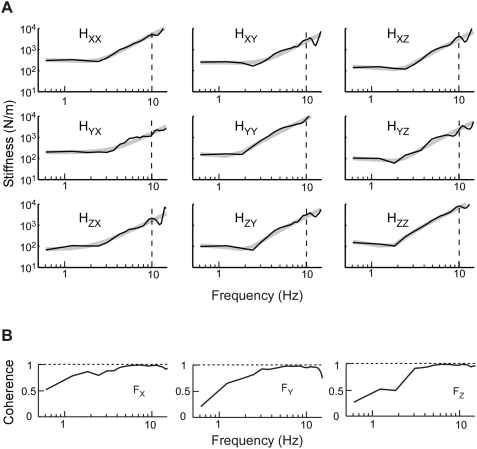
Nonparametric estimates of endpoint impedance for a single experimental condition. The subject's posture placed the hand at 190 mm in front of the sternum and had the shoulder abducted to 14°. (A) Nonparametric transfer functions (gray lines) and the corresponding second-order fits (black lines). (B) Multiple coherence functions for forces along each of the three measurement axes. Horizontal dashed lines (1.0) correspond to perfect coherence.

Linear, nonparametric transfer functions were appropriate for characterizing the 3D impedance of the human arm. Figure 7A illustrates the magnitude portion of typical endpoint impedance transfer functions. Across all subjects and bias forces, the average R^2^ value for force data predicted by these transfer functions was 92.6±1.1%. Additionally, the multiple coherence functions for each output approached 1.0 between ∼2–10 Hz. (Figure 7B). This result was consistent across subjects and indicated that for these frequencies the recorded endpoint forces were well described using a linear model. Finally, these nonparametric transfer functions (Figure 7A; black lines) were well characterized by second-order models (Figure 7A; gray lines) over the range of 0–10 Hz. These parametric models described 71.4±2.0% of the data variance over this frequency range.

At each posture, stiffness was estimated as subjects exerted 4 levels of voluntary force. The results from all force levels at a given posture were averaged to provide a single estimate of stiffness orientation. This was possible because the small bias forces used in this study did not cause consistent changes in the orientation of maximal endpoint stiffness. This was assessed using an ANOVA to compare the influence of endpoint force on the estimated orientation of maximal endpoint stiffness. Separate ANOVAs were performed for each posture. In all cases, the influence of force was not significant (all p> = 0.30). It is important to note that the endpoint force targets along each of the measurement axes contributed to only a fraction of the muscle activity required to complete the postural tasks in this study. Subjects also were required to support the weight of their limb in all experiments and the muscle activity required to accomplish that goal likely dominated the measured endpoint stiffness. When subjects do not support the weight of their limb, it is well documented that small changes in endpoint force can have significant effects on the orientation of maximal endpoint stiffness [17], [26], [27].

### Influence of Posture on Direction of Maximal Endpoint Stiffness

**Figure 8:**
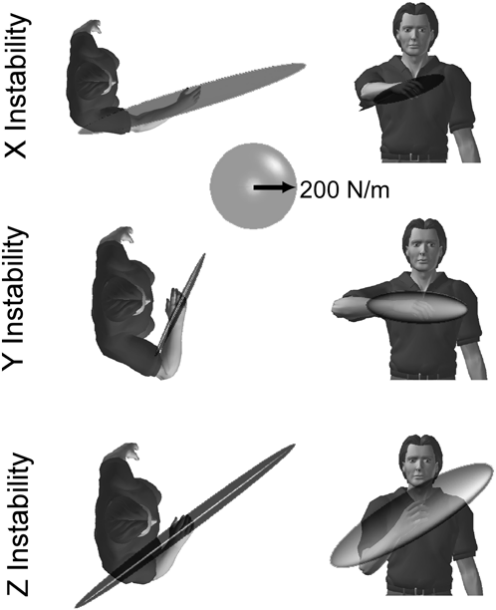
Endpoint stiffness ellipsoids from a typical subject. Two views are shown for the self-selected postures used for each haptic environment. At each posture, stiffness was estimated as the subject applied a +10 N force along the direction of the haptic instability.

The self-selected postures for each environment tended to orient the direction of maximal stiffness toward the direction of the environmental instability. This can be seen in Figure 8, which displays typical endpoint stiffness ellipsoids overlaid on characters that depict the posture selected by this subject. For example, during interactions with the Y instability this subject positioned the forearm in the horizontal plane and the hand close to the sternum. At this posture, maximal stiffness was oriented primarily in the horizontal plane, rotated toward the Y axis (Figure 8, middle row). For interactions with the Z instability, the shoulder abduction angle was small, which had the effect of rotating the direction of maximal stiffness vertically, toward the Z axis (Figure 8; third row). This subject extended the arm during interactions with the X instability, which tended to orient the direction of maximal stiffness towards the X axis (Figure 8; top row).

**Figure 9:**
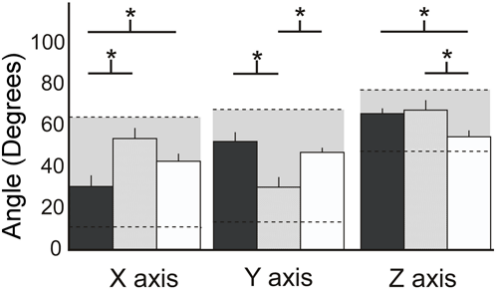
Orientation of maximal endpoint stiffness for each self-selected posture. Each bar corresponds to the orientation of maximal endpoint stiffness relative to the orientation of the X, Y, and Z axes. The dark gray bars correspond to postures selected during interactions with the haptic environment that was unstable in the X direction; the light gray bars correspond to postures selected during interactions with the haptic environment that was unstable in the Y direction; the white bars correspond to postures selected during interactions with the haptic environment that was unstable in the Z direction; The asterisks above the bars correspond to significant differences between stiffness orientations across the self-selected postures (p<0.01; paired t-test). Bars are mean±standard error. The horizontal dashed lines denote the range of possible stiffness orientations.

These results were consistent across the group of subjects tested. This was examined by calculating the angles between the major axis of the estimated stiffness ellipsoids and the vector describing the X, Y and Z axes, along which the three unstable environments were aligned (Figure 9). For each self-selected posture, the corresponding orientation of maximal endpoint stiffness was most closely aligned with the orientation of the environmental instability with which the subject was interacting (paired t-test; all * in Figure 9 denote p<0.001).

The direction of maximal endpoint stiffness for each self-selected posture was never perfectly aligned with the direction of the environmental instability. Such an alignment was not possible given the experimental constraint of keeping the hand in front of the sternum. The available range of stiffness orientations was measured in two subjects, as described previously. The limits of this range across both subjects are indicated by the dashed lines in Figure 9. Note that the range of available stiffness orientations was consistent across both subjects. Across all postures, the average difference in the orientation of maximal endpoint stiffness between subjects was only 4±1°.

### Controlling for Forearm Rotation

Rotation of the forearm did not influence estimates of maximal stiffness orientation. Since the forearm postures selected during the tracking experiments did not necessarily match those during the estimation of arm stiffness, we performed a control experiment to determine the influence of forearm rotation on endpoint stiffness orientation. Posture dependent changes in stiffness were examined using a linear mixed-effects model to examine changes in stiffness orientation. The independent factors were forearm posture and the level of voluntary force; subjects were treated as a random factor, and the dependent factors were the angles of the stiffness orientation vector projected into the XY and YZ planes. For both projections, the influence of posture was small. The largest change in stiffness orientations between the tested postures was 1.97±1.33°, which was between the most supinated and pronated positions, This effect did not reach statistical significance (t = 1.47, p = 0.15) and was small relative to the changes due to variations in hand distance and shoulder abduction angle (Figure 9).

## Discussion

The purpose of this study was to determine how subjects compensate for environmental instabilities during tasks in which they are free to select from a range of available arm postures. First, we tested the influence of different prescribed postures on the ability to maintain endpoint position within directionally unstable environments. This performance was then tested against that obtained at the self-selected postures. Importantly, posture significantly influenced performance, and performance was always best at the self-selected posture. When posture had a large influence on performance, subjects self-selected similar postures that tended to orient the direction of maximal endpoint stiffness toward the direction of the environmental instability. Limb mechanics, which were quantified using estimates of endpoint stiffness, can be regulated using a variety of motor behaviors. Our results suggest that when the arm is unconstrained, posture selection is a fundamental means by which these mechanics are regulated.

### Influence of Posture on Tracking Performance

Posture had a strong influence on the ability to maintain stable endpoint locations in our experiments. The present results are consistent with those reported by Milner [15], who examined the influence of arm posture on the ability to maintain endpoint location during interactions with unstable loads oriented within the horizontal plane. In his studies, subjects attempted to hold the hand at the equilibrium position of the unstable environments using two prescribed postures. During interactions with an environment that was unstable in the medial-lateral direction, task performance was greatly increased when the hand was held near the trunk, relative to when it was held away from the body. Also, during interactions with loads in the anterior-posterior direction, task performance was only slightly affected by posture. In both cases, it appeared likely that postural changes had a stronger influence on the ability to maintain stable endpoint postures than changes in voluntary co-contraction. Our results are similar. In addition, we extended Milner's findings to 3D and demonstrated that when the limb is unconstrained, subjects self-select postures that increase limb stability and the corresponding ability to maintain steady hand positions in the presence of destabilizing environmental loads.

### Regulation of Limb Mechanics

In addition to improving tracking performance, the self-selected postures tended to orient the direction of maximal stiffness toward the direction of the environmental instability. These results support the idea that endpoint stiffness is regulated to counteract environmental instabilities and improve task performance [1], [3], [28] and that this regulation can occur, at least partly, through voluntary changes in limb posture. Additional motor behaviors also may have been used to improve performance during the tracking tasks. The most obvious is voluntary co-contraction, which can be used to increase limb stiffness and to provide stability during interactions with destabilizing environments [6], [29]. However, co-contraction is metabolically costly, and it has been suggested that humans tend to use the minimal amount of co-contraction necessary to maintain limb stability [30]. It is likely that subjects in the present study also attempted to minimize co-contraction, and that this minimization was done by selecting a limb posture that matched the intrinsic mechanical properties of the arm to the functional requirements of the task. By relying more heavily on postural shifts than voluntary co-contraction, it may be possible to decrease energy expenditure. This notion is consistent with studies suggesting that the central nervous system attempts to minimize energy expenditure during unconstrained tasks [31], [32].

Increased stretch reflex sensitivity also may have influenced tracking performance during interactions with the unstable loads. Stretch sensitive reflexes are known to modulate with changes in the mechanical properties of the environment [7], [8], [10] and can substantially alter the mechanical properties of a limb [33]–[36]. Furthermore, it has been suggested that adaptation of reflex gains can provide a means for maintaining limb stability while minimizing energy expenditure [37], [38]. Such an optimization may have occurred in these experiments, but if so, it was done in concert with the observed postural shifts.

We have described our results primarily in the context of endpoint stiffness. However, posture also has a strong effect on the inertial and viscous properties of the arm [2], [18], [39], and any of these posture dependent changes in arm mechanics and use may have influenced tracking performance. We have focused on endpoint stiffness because we examined the performance of a postural task and because the task involved interactions with negative stiffness fields. Nevertheless, contributions from other components of limb impedance would still support our conclusion that when the limb is unconstrained, subjects self-select arm postures that match the mechanical properties of the arm to the mechanical constraints of the task.

The force manipulability [40] of a limb changes with posture in a manner that is similar to endpoint stiffness, and it is possible that subjects chose postures that were optimal for generating forces required to maintain the endpoint at the target locations. While our current data do not allow us to definitively rule out this possibility, we consider it to be unlikely that postures were selected to optimize only force manipulability. The maximum forces due to the environmental instability at each of these targets was only 5 N, which is small relative to the maximum forces that could be generated and, more importantly, small relative to the equivalent endpoint force required to support the weight of the limb during these tasks. The equivalent endpoint force required to oppose gravity would be approximately 15 N in the vertical direction for a typical 70 kg subject [41]. Therefore, during interactions with all environments, the largest component of the endpoint force, and the associated muscle activity required to complete the task, was in the vertical direction. Nevertheless, there were substantial changes in the self-selected postures during interactions with each of the haptic environments. Only during interactions with the Z instability would these postures have resulted in near-optimal force manipulability [42]. This is the one condition in which the endpoint force required for the task was co-aligned with the haptic instability.

### Consistency of Self-Selected Postures

Consistent postures were selected only when arm posture had a substantial effect on task performance. For example, when interacting with the Z instability, both hand location and shoulder abduction had large effects on the ability to maintain stable hand positions. All subjects performing this task selected consistent hand and shoulder postures that enhanced tracking performance. In contrast, hand position had the largest influence on tracking ability when interacting with the Y instability, and this is the degree of freedom that was most consistently selected by the subjects. Much more variability was seen in the shoulder angles selected by all. Posture had the least influence on tracking when subjects interacted with the X instability. Correspondingly, the self-selected postures were most variable across both degrees of freedom when subjects interacted with this environment. These posture selection results are consistent with the concept of controlling degrees of freedom most relevant to task performance [43], [44].

Task performance was not greatly affected by arm posture when subjects interacted with the X instability. This is somewhat counterintuitive since it is well documented that arm stiffness can be dramatically increased along this direction by reaching outward from the body [2], [17], [26]. These changes in arm stiffness, which would have occurred for the most extended prescribed postures, had little effect on tracking performance. This most likely is due to the fact that endpoint stiffness in these tasks was limited more by the trunk than by the arm [19]. Due to the fact that subject were required to have a side-by-side stance for these experiments, trunk compliance likely would have been lowest in the X direction and therefore would have had the greatest influence on the mechanical properties of the endpoint for tasks in which this direction was most relevant. Though we were most interested in the relationship between arm mechanics and task performance, this result emphasizes that during natural tasks the mechanics and posture of the entire body can contribute to the endpoint mechanics of the arm.
